# Noradrenergic Pheochromocytoma: A Case Report

**DOI:** 10.7759/cureus.27492

**Published:** 2022-07-30

**Authors:** Mohammed Amine Essafi, Sanae Habibi, Hayat Aynaou, Houda Salhi, Hanan El Ouahabi

**Affiliations:** 1 Department of Endocrinology, Diabetology, Metabolic Diseases and Nutrition, Hassan II University Hospital Center, Fez, MAR

**Keywords:** normetanephrine, adolescent, adrenals, pheochromocytoma, paraganglioma

## Abstract

Pheochromocytomas and paragangliomas are rare neuroendocrine tumors developed from chromaffin cells. They are exceptional in children with an atypical symptomatology. We report here a case of a 17-year-old boy who presented with a left retroperitoneal mass discovered on thoracic-abdominal-pelvic computed tomography (CT) scan in the presence of diffuse abdominal pain, more pronounced in the left hypochondrium. The exploration had objectified an exclusive secretion of urinary normetanephrine over 24 hours. Metanephrine and 3 ortho methyldopamine were within normal limits. He had adrenalectomy after controlling his blood pressure with an alpha blocker. Histology had confirmed a pheochromocytoma of non-aggressive potential *Pheochromocytoma* of the Adrenal gland Scaled *Score *(PASS) 2. The evolution was favorable with normalization of blood pressure and urinary catecholamines at one week, three months, six months, and one year. He tested negative for hereditary syndromic including Von Hippel-Lindau (VHL), RET genes, subject to the succinate dehydrogenase complex B and D subunit genes (SDHB-D), which have been requested.

## Introduction

Pheochromocytomas and paragangliomas are rare tumors that develop from chromaffin cells, producing an excess of catecholamines [1]. Their main clinical manifestation is hypertension. They are less frequent in children than in adults, with an estimated incidence of 0.11 per million children [2].

There is a variation in catecholamine secretory phenotype between pheochromocytomas and paragangliomas, with primary or exclusive normetanephrine secretion in paraganglioma, whereas metanephrine secretion is usually confined to pheochromocytomas originating from the adrenal medulla [3].

Here we report a case of pheochromocytoma in a 17-year-old adolescent with excess normetanephrine but normal metanephrine levels.

## Case presentation

**Figure 1:**
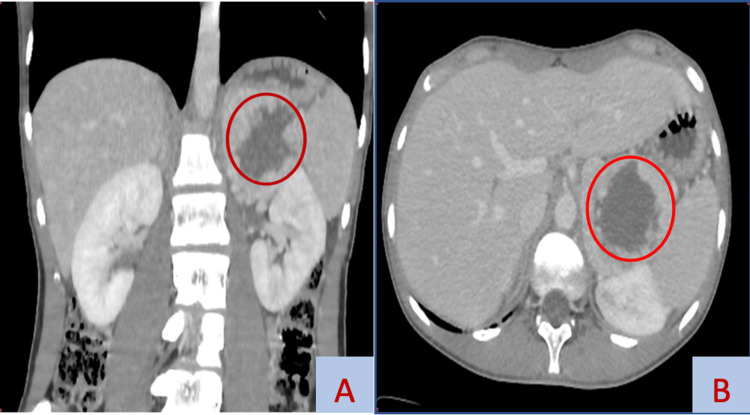
Frontal (A) and transverse (B) abdominal CT sections showing a 60 x 72 x 75 mm tissue mass between the spleen and peritoneum with a necrotic appearance (red circle).

A 17-year-old adolescent presented with an incidentally discovered left retroperitoneal mass measuring 6 × 7.2 × 7.5 cm on a thoracic-abdominal-pelvic computed tomography (CT) scan performed in the face of diffuse abdominal pain that was more pronounced in the left hypochondrium (Figure 1).

The meticulous interrogation had objectified paroxysmal crisis made of profuse sweating, headaches, and palpitations evoking a triad of Menard associated with neurosensory signs of arterial hypertension in the form of tinnitus. On admission, the patient presented with a discharge crisis with high blood pressure, around 200/120 mmHg, and a heart rate of 115 beats per minute, having ceded spontaneously after 10 minutes with normalization of the blood pressure without orthostatic hypotension. The rest of the clinical examination was unremarkable.

Complementary examinations revealed an elevation of normetanephrine in the 24-hour urine (4,050 nmol/L or 8.9 x normal). Metanephrine and 3 ortho methyldopamine in 24-hour urine were within normal limits. The workup for extension and secondary localization was unremarkable.

The patient benefited during 10 days from a preparation to the surgery by an alpha blocker prazosin to control his arterial pressure, introduction of a calcic inhibitor after 7 days, and a good hydration to increase the blood volume and avoid postsurgical hypotension. A preoperative embolization of the superior and inferior adrenal arteries was performed. The procedure was uneventful.

The patient underwent a left adrenalectomy, with the notion of three adrenergic crisis during the surgical procedure. The post-surgery course was uneventful.

His blood pressure was stable after the surgery. Histology had confirmed the diagnosis of pheochromocytoma of non-aggressive potential (PASS score 2). The evolution was favorable with normalization of blood pressure and urinary methoxylated derivatives at one week, three months, six months, and one year after surgery (Table 1).

**Table 1:** Evolution of urinary catecholamines at diagnosis and after surgery. N: normal

	At diagnosis	1 week after surgery	3 months	6 months	1 year
Normetanephrine (nmol/L)	4,050 or 8.9 x normal	79	70	76	72
Metanephrine (nmol/L)	35	32	32	33	30
3 ortho methyldopamine (nmol/L)	170	150	148	150	132

As part of the genetic syndromic workup, the search for genetic mutations including Von Hippel-Lindau (VHL) and RET genes were negative; the B and D genes of the succinate dehydrogenase complex (SDHB-D) are still in progress.

## Discussion

Pheochromocytomas and paragangliomas are rare neuroendocrine tumors. Although widely described in the adult literature, in the pediatric population pheochromocytomas remain less frequent and poorly described [2].

The clinical presentation is atypical. Excess of secreted catecholamines lead to hypertension, excessive sweating, and tachycardia. Other symptoms may be present, such as abdominal pain.

Paragangliomas primarily secrete normetanephrine, whereas metanephrine secretion is usually limited to pheochromocytomas arising from the adrenal medulla [3]. One hypothesis that explains this secretory phenotype is the proximity of pheochromocytomas (originating from the adrenal medulla) to cortical steroids that produce the enzyme phenylethanolamine-N-methyltransferase that converts norepinephrine to epinephrine [4].

Our patient had an adrenal pheochromocytoma producing exclusively normetanephrine. One study found that noradrenergic tumors generally exhibit continuous hypertension, and metanephrine-producing tumors cause paroxysmal hypertension [5], which is in contrast to the case of our patient, who had paroxysmal hypertension with exclusively noradrenaline secretion.

More than 40% of affected patients have identifiable germline mutations; therefore, international guidelines suggest genetic testing for all patients regardless of age [6-7]. The most frequently tested genes are VHL, RET, SDHD, and SDHB [8]. In our patient, he was considered negative for the hereditary gene mutations associated with pheochromocytoma, suggesting a sporadic type subject to the succinate dehydrogenase complex B and D subunit genes (SDHB-D), which are still in progress.

Surgical resection is the treatment of choice, along with pre-operative preparation to prevent cardiovascular complications by alpha-blockers, calcium channel blocker, and good hydration to prevent severe hypotension after tumor removal [9]. Open surgery is recommended for tumors larger than 6 cm because it minimizes catecholamine release and allows complete resection [10]. Our patient underwent left adrenalectomy by open surgery.

## Conclusions

Pheochromocytoma and paraganglioma are rare tumors in the pediatric population. Their diagnosis is suspected in the presence of an adrenal mass or hypertension and is based on the determination of urinary catecholamines. Paragangliomas primarily secrete normetanephrine, whereas metanephrine secretion is usually limited to pheochromocytomas. Our case report shows an adrenal pheochromocytoma producing exclusively normetanephrine. The management is medical-surgical and multidisciplinary. Radical resection is the treatment of choice. Genetic testing is important in patients with pheochromocytoma/paraganglioma.
